# Comparison of Administration Routes on the Protective Effects of Bee Venom Phospholipase A2 in a Mouse Model of Parkinson’s Disease

**DOI:** 10.3389/fnagi.2018.00179

**Published:** 2018-06-11

**Authors:** Hyunjung Baek, Hyun Il Jang, Hat Nim Jeon, Hyunsu Bae

**Affiliations:** Department of Physiology, College of Korean Medicine, Kyung Hee University, Seoul, South Korea

**Keywords:** Parkinson’s disease, bee venom phospholipase A2 (bvPLA2), route of administration, tyrosine hydroxylase, regulatory T cells (Tregs)

## Abstract

Parkinson’s disease (PD) is the second most common neurodegenerative disorder worldwide. Progressive loss of dopaminergic neurons in the substantia nigra (SN) and their synaptic terminal connections in the striatum are main characterizations of PD. Although many efforts have been made to develop therapeutics, no treatment has been proven effective. We previously demonstrated that bvPLA2 can protect dopaminergic neurons by modulating neuroinflammatory responses in an MPTP (1-methyl-4-phenyl-1,2,3,6-tetrahydropyridine)-induced mouse model of PD. The cellular basis for the neuroprotective response of bvPLA2 was the induction of CD4^+^CD25^+^ regulatory T cells (Tregs), a population known to suppress immune activation and maintain homeostasis and tolerance to self-antigen. The aim of the present study was to investigate the effects of different routes of bvPLA2 administration in a PD mouse model. Neurobehavioral assessment revealed progressive deterioration in locomotor functions of the MPTP group compared with the control group. However, such functions were improved following subcutaneous (s.c.) bvPLA2 administration. The results showed that the s.c. route of bvPLA2 administration contributed to the induction of Treg cells and the reduction of Th1 and Th17 populations, demonstrating that the neuroprotective effects were associated with reduced tyrosine hydroxylase (TH)-positive dopaminergic neurons and microglia. These results suggested that the s.c. bvPLA2 injection could be beneficial for treating aspects of PD.

## Introduction

Parkinson’s disease (PD) is a common degenerative disease of the central nervous system that affects motor functions, such as muscular rigidity, onset of tremor, slowness of voluntary movements and difficulty maintaining balance. PD is caused by the progressive loss of dopaminergic neurons in the substantia nigra (SN). Current symptomatic therapy for PD can provide a benefit to slow disease progression and may cause motor complications, such as motor fluctuation and dyskinesia. Therefore, therapeutic strategies to delay the onset or slow progression of PD are needed. Functional links between adaptive immunity and neurodegenerative disorders have been well studied for PD, Alzheimer’s disease (AD) and multiple sclerosis (Bar-Or et al., 2003; Casal et al., 2003; Baba et al., 2005). A specific population of T cells currently recognized as CD4^+^CD25^+^Foxp3^+^ regulatory T cells (Tregs) suppress immune activation and maintain immune homeostasis and tolerance. Tregs mediate neuroprotection through the modulation of inflammatory responses, suppression of microglial activation, and promotion of neuronal survival in animal models of PD (Reynolds et al., 2007, 2010). Therefore, the development of fast and effective methods of generating Tregs is likely to play a key role in the development of therapies for PD patients.

Bee venom (BV) extracted from honeybees is commonly used in Korean medicine. Previous studies have reported that BV therapy has anti-nociceptive and anti-inflammatory effects on pain, arthritis, cancer and skin disease (Kwon et al., 2001, 2002). Recent studies suggest that BV treatment possesses beneficial effects on the loss of dopaminergic neurons in PD and motor neurons in amyotrophic lateral sclerosis (ALS; Yang et al., 2010; Kim et al., 2011). BV therapy can be used in a cream, liniment, ointment, via injection, acupuncture or even direct administration of live bee stings. The most commonly used method is BV acupuncture, which involves a subcutaneous injection of diluted BV into acupoints. BV acupuncture has long been employed for numerous disorders including arthritis, pain syndrome, and multiple sclerosis (Shinto et al., 2004; Kim et al., 2005; Lee et al., 2005). Recently, several randomized controlled trials (RCTs) patients with idiopathic PD evaluated the safety and efficacy of BV therapy, including BV acupuncture and subcutaneous injection (Cho et al., 2012; Doo et al., 2015; Hartmann et al., 2016). Considering the findings reported on BV therapy for PD, the different efficacy reported to date may be due to the therapeutic protocol used, the severity of PD, in addition to potential time and dose-dependent properties. Interestingly, another recently published study compared the effects of BV treatment using different administration routes for the symptomatic hSOD1^G93A^ transgenic model of ALS (Cai et al., 2015). Taken together, these findings are requested further well designed RCTs for the therapeutic potential of BV treatment, including the most effective doses and mode of administration, and comparison its effects in different stages of PD patients.

BV contains several bioactive compounds, including melittin, phospholipase A2 (PLA2), apamin and peptides (Lariviere and Melzack, 1996). Studies have shown that bvPLA2 possesses protective effects by inducing Treg populations in neurodegenerative diseases, such as AD and PD murine models (Chung et al., 2015; Ye et al., 2016). Chung et al. (2015) reported the neuroprotective effects of bvPLA2 in controlling the generation of Tregs in MPTP-induced mouse model of PD. These authors demonstrated that bvPLA2 could promote the survival of dopaminergic neurons by suppressing microglial activation and reducing the infiltration of CD4^+^ T cells in a PD mouse model. However, it is unclear which route of bvPLA2 administration would be more relevant for the treatment of PD. Therefore, the purpose of the present study was to investigate which route of bvPLA2 administration would be more effective for protecting dopaminergic neurons in MPTP-induced PD models. Intraperitoneal (i.p.), subcutaneous (s.c.), intramuscular (i.m.) and intravenous (i.v.) injection routes were examined. The findings highlight the optimum route of bvPLA2 administration to exert its neuroprotective effects on MPTP-induced PD in a mouse model by controlling the generation of Treg populations.

## Materials and Methods

### Animals

All experiments were performed in accordance with the approved animal protocols and guidelines established by Kyung Hee University. Seven to eight-week-old male C57BL/6J mice (19–23 g) were purchased from The Jackson Laboratory (Bar Harbor, ME, USA). The mice were maintained on a 12 h light/dark cycle and temperature-controlled conditions (21 ± 2°C), with food and water *ad libitum*.

### Animal Experiments

For MPTP intoxication, the mice received four i.p. injections of MPTP-HCl (20 mg/kg free base in saline; Sigma-Aldrich, St. Louis, MO, USA) at 2-h intervals as previously described (Chung et al., 2011). Twelve hours after the last MPTP injection, the mortality rate of mice was 30.4%. Then, live mice were divided into five groups and received i.p., s.c., i.m., or i.v. injections of bvPLA2 (0.5 mg/kg) for 6 days. Some mice were injected with vehicle alone as controls.

### Measurement of Motor Activity by Pole Test

Twenty-four hours after the last bvPLA2 injection, a pole test was performed to determine forelimb and hindlimb motor coordination and balance. Briefly, the mice were place on the top of a gauze-banded wooden pole (50 cm in length and 0.8 cm in diameter) facing upwards. The animals were allowed to climb down to the base of the pole. The time to turn completely downward (T-turn) and the total time taken for the mouse to reach the floor (locomotion activity time [T-LA]) were recorded, with a cut-off limit of 30 s. Each mouse was examined in five trials, and the average of the best three measurements was calculated. Trials with the mouse jumping or sliding down the pole were excluded.

### Flow Cytometric Analysis of Th1, Th2, Th17 and Treg Populations

Flow cytometric analysis of T-helper cell subsets was performed by using fluorescein isothiocyanate (FITC)-conjugated anti-mouse CD4 (clone GK1.5; eBioscience, San Diego, CA, USA), phycoerythrin (PE)-conjugated anti-mouse CD25 (clone PC61.5; eBioscience), Alexa Fluor 647 anti-mouse/rat Foxp3 (clone MF23; BD Biosciences), PE-conjugated anti-mouse IFN-γ (clone SMG1.2; eBioscience) PE-conjugated anti-mouse IL-4 (clone 11B11; eBioscience), and PerCP-Cyanine5.5-conjugated anti-mouse/rat IL-17A (clone eBio17B7; eBioscience) antibodies. For Treg staining, single-cell splenocytes at a concentration of 2 × 10^6^ cells/ml were collected, washed twice with phosphate-buffered saline (PBS), and stained with FITC-conjugated anti-CD4 and PE-labeled anti-CD25 antibodies in staining buffer for 30 min on ice in the dark. The cells were subsequently washed twice with PBS and fixed in fixation/permeabilization buffer (eBioscience) for 1 h at 4°C in the dark. Subsequently, the cells were washed twice with PBS and stained with Alexa Fluor 647 anti-Foxp3 antibody overnight at 4°C in the dark. After washing, the cells were fixed in 1% paraformaldehyde and stored at 4°C in the dark for subsequent detection. For intracellular cytokine staining, single-cell splenocytes at a concentration of 2 × 10^6^ cells/ml were stimulated with 50 ng/ml of phorbol myristate acetate (PMA; Sigma-Aldrich) and 1000 ng/ml of ionomycin (Sigma-Aldrich) for 1 h, and then incubated with GolgiStop (BD Biosciences) for 4 h. The cells were collected, washed twice with PBS, and stained with FITC-labeled anti-CD4 antibody for 30 min on ice in the dark. After washing with PBS, the cells were fixed in IC fixation buffer (BD Biosciences) for 30 min at room temperature in the dark. The cells were then stained with PE-conjugated anti-IFN-γ, PE-conjugated anti-IL-4 and PerCP-Cyanine5.5-conjugated anti-IL-17A antibodies overnight at 4°C (Lee et al., 2016). After washing with PBS, the cells were fixed in 1% paraformaldehyde and stored at 4°C in the dark for subsequent detection. After the samples were analyzed with a FACSCalibur flow cytometer (BD Biosciences), the data were generated in graphical and tabular formats by using FLOWJO software (Tree star, Ashland, OR, USA).

### Tissue Processing and Immunohistochemistry

The mice were anesthetized with isoflurane (Forane solution; ChoongWae Pharma, Seoul, South Korea) and transcardially perfused with PBS followed by perfusion with a fixative solution containing 4% paraformaldehyde dissolved in 0.1 M phosphate buffer. The brain was dissected, postfixed in 4% paraformaldehyde at 4°C overnight, transferred to 30% sucrose solution, and subsequently frozen. The tissues were embedded in OCT compound and serially cut on a cryostat into 30-μm thick coronal sections by using a sliding microtome. All sections were collected in six separate series and processed for immunohistochemical staining. Primary antibody was directed against tyrosine hydroxylase (TH; 1:2000, Pel-Freez Clinical System, Brown Deer, WI, USA) and Iba-1 (1:2000), Wako Pure Chemic Industries, Osaka, Japan). After washing with PBS, the sections were incubated with the appropriate biotinylated secondary antibody and processed with an avidin-biotin complex kit (Vectastain ABC kit; Vector Laboratories, Burlingame, CA, USA). The reaction product was visualized with 0.05% diaminobenzidine-HCL (DAB) and 0.003% hydrogen peroxide in 0.1 M phosphate buffer. The labeled tissue sections were subsequently mounted and analyzed under a bright field microscope (Nikon, Tokyo, Japan).

### Stereological Cell Counts

Unbiased stereological estimation of the total number of TH-positive dopaminergic neurons in the SN was performed by using an optical fractionator method on an Olympus CAST (computer-assisted stereological toolbox) system version 2.1.4. (Olympus, Ballerup, Denmark) as previously described (Chung et al., 2011). Sections used for counting covered the entire SN from the rostral tip of the pars compacta to the caudal end of the pars reticulate (anteroposterior, from −2.06 mm to −4.16 mm from bregma). Actual counting was performed by using a 100× oil objective. The total number of cells was estimated according to the optical fractionator equation. More than 300 points were analyzed for all sections of each specimen.

### Statistical Analysis

All data were analyzed using GraphPad Prism 5.01 (GraphPad Software Inc., San Diego, CA, USA). The data are presented as the means ± standard error of the mean (SEM) where indicated. Statistical significance of each variable was evaluated by one-way analysis of variance (ANOVA), followed by Newman-Keuls multiple comparison test for multiple comparison or by two-tailed Student’s *t*-test for single comparisons. All experiments were performed in a blind manner and repeated independently under identical conditions. Statistical significance was set at *P* < 0.05.

## Results

### Effects of Different Routes of bvPLA2 Administration on Motor Function in MPTP-Induced Neurotoxicity

**Figure 1:**
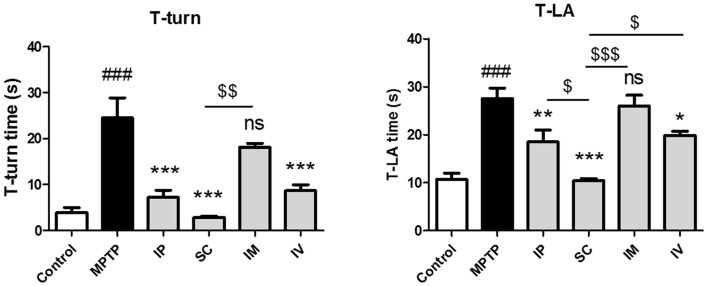
Effects of the administration routes of Bee Venom Phospholipase A2 (bvPLA2) on T-turn and T-total time values in (1-methyl-4-phenyl-1,2,3,6-tetrahydropyridine; MPTP)-injected mice. The pole test was performed at 24 h after the last bvPLA2 injection. Behavioral function was improved when bvPLA2 was subcutaneously administered. Error bars represent the means ± standard error of the mean (SEM). The significance was determined by Student’s *t*-test. ^###^*P* < 0.001 vs. the respective phosphate-buffered saline (PBS) control. *P < 0.05, **P < 0.01, ***P < 0.001, significantly different from the substantia nigra (SN) of MPTP-injected mice. ^$^*P* < 0.05, ^$$^*P* < 0.01, ^$$$^*P* < 0.001 vs. the SC group. IP, intraperitoneal injection; SC, subcutaneous injection; IM, intramuscular injection; IV, intravenous injection.

To evaluate the effects of different routes of bvPLA2 administration on MPTP-induced neurotoxicity, mice were administered MPTP and received a single daily injection via i.p., s.c., i.m. and i.v. of bvPLA2 or saline for 6 days commencing 12 h after the last MPTP injection. Various behavioral tests have been routinely used to qualify PD mouse models, including locomotor activity, rotarod test, forepaw stride length, and the pole test (Taylor et al., 2010). In the present study, the neuroprotective effect of bvPLA2 against PD-related motor deficits was evaluated by the pole test in an MPTP-induced mouse model. MPTP treatment significantly extended the time of T-turn and T-LA compared to those in the control group (Figure 1). T-turn and T-LA were significantly shortened in the SC group compared to those in the MPTP group. IP and IV administration of bvPLA2 showed moderate recovery of motor coordination and balance. However, there were no significant differences in the time of T-turn and T-LA between the MPTP and IM groups.

### Effects of Different Routes of bvPLA2 Treatment on Treg Populations in MPTP-Injected Mice

**Figure 2:**
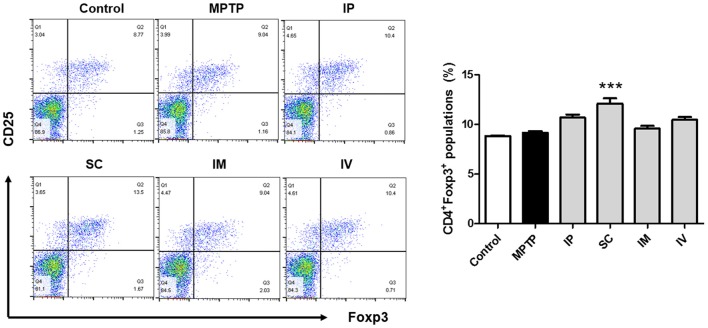
Differentiation of Treg cells induced by bvPLA2 depending on routes of administration for MPTP-injected mice. Treg differentiation in splenocytes was assessed by flow cytometry for cells expressing CD4, CD25 and Foxp3. The bar graph depicts the statistics of Tregs in splenocytes. The number of Treg cells was increased when bvPLA2 was subcutaneously administered. Error bars represent the means ± SEM. The significance was determined by Student’s *t*-test. ***P < 0.001, significantly different from MPTP-injected mice. IP, intraperitoneal injection; SC, subcutaneous injection; IM, intramuscular injection; IV, intravenous injection.

In a previous report, the proportion of Treg cells was decreased in PD patients compared to that in the control group (Chen et al., 2015). Furthermore, the impaired ability of Tregs from PD patients to suppress effector T cell functions was observed. The effects of different injection routes of bvPLA2 on the proportions of Tregs were assessed by flow cytometric analysis. No significant difference in the CD4^+^CD25^+^Foxp3^+^ Treg populations was detected between the control and MPTP groups. However, a more than 30% increase in the number of Treg cells was detected in the SC group compared to that in the control group (Figure 2). The other routes of bvPLA2 injection did not induce any significant difference in the number of Tregs within this population.

### Effects of Different Routes of bvPLA2 Treatment on Th1/2/17 Populations in MPTP-Induced PD Mice

**Figure 3:**
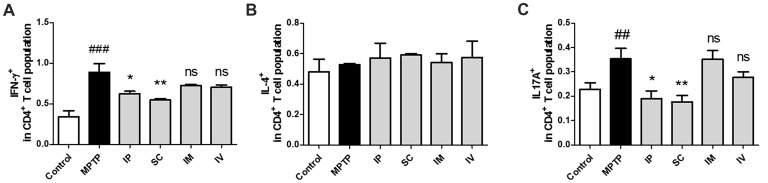
Differentiation of Th1/2/17 cells induced by bvPLA2 depending on the routes of administration to MPTP-injected mice. Th1/2/17 differentiation in splenocytes was assessed by flow cytometry for **(A)** Th1 (CD4^+^IFN-γ^+^), **(B)** Th2 (CD4^+^IL-4^+^) and **(C)** Th17 (CD4^+^IL-17A^+^) populations. The bar graph depicts the statistics of Th1 cells in splenocytes. The error bars represent the means ± SEM. The significance was determined by Student’s *t*-test. ^##^*P* < 0.01 and ^###^*P* < 0.001 vs. the respective PBS control. *P < 0.05 and **P < 0.01, significantly different from MPTP-injected mice. IP, intraperitoneal injection; SC, subcutaneous injection; IM, intramuscular injection; IV, intravenous injection.

The effects of different routes of bVPLA2 administration on T-helper cell phenotypes were subsequently determined. Changes in IFN-γ, IL-4 and IL-17A expression suggest an alternative status of Th1, Th2 and Th17 cells. The prevalence of these cells was then further determined by flow cytometric analysis. As shown in Figure 3A, the Th1 population was increased and the Th1/Th2 balance was shifted toward Th1 in the MPTP group, indicating an enhanced Th1-type response. The present study also found that Th17 cells were increased in the MPTP group. Compared to the MPTP group, flow cytometric analysis indicated that polarized Th1 and Th17 cells showed the decreased IFN-γ and IL-17A expression in the IP and SC group (Figure 3C). No significant differences were detected for Th2 polarization between all groups (Figure 3B).

### Effects of Routes of bvPLA2 Administration on Dopaminergic Neurons in the SN Against MPTP Neurotoxicity

**Figure 4:**
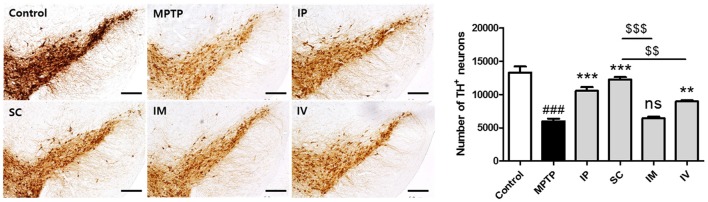
Immunohistochemical staining for tyrosine hydroxylase (TH) in striatum tissue samples from bvPLA2-administered groups by different routes. Brain tissues were stained with TH for the analysis of damage on dopaminergic neurons. The number of TH-positive neurons in the SN was quantified by using a semi-quantitative scale. All randomly selected histological images were scored. These data are representatives of three separate experiments. Data are shown as the means ± SEM. ^###^*P* < 0.001 vs. the CON group, **P < 0.01 and ***P < 0.001 vs. the MPTP group, and ^$$^*P* < 0.01 and ^$$$^*P* < 0.001 vs. the SC group. Scale bars: 5.0 μm. IP, intraperitoneal injection; SC, subcutaneous injection; IM, intramuscular injection; IV, intravenous injection.

To assess the protective effect of each administration route of bvPLA2 on dopaminergic neurons, TH-positive neurons were counted (Figure 4). Treatment with MPTP reduced the number of TH-positive neurons more than two-fold compared to that in the control group. However, analysis of surviving dopaminergic neurons in TH-immunostained striatum after MPTP and bvPLA2 treatments revealed that i.p., s.c., and i.v. injections of bvPLA2 increased the number of surviving TH-positive neurons within the SN, whereas i.m. injection of bvPLA2 remarkably diminished dopaminergic neurons within the striatum (Figure 4).

### Effects of Routes of bvPLA2 Injection on Microglial Activation From MPTP-Derived Neurotoxicity

**Figure 5:**
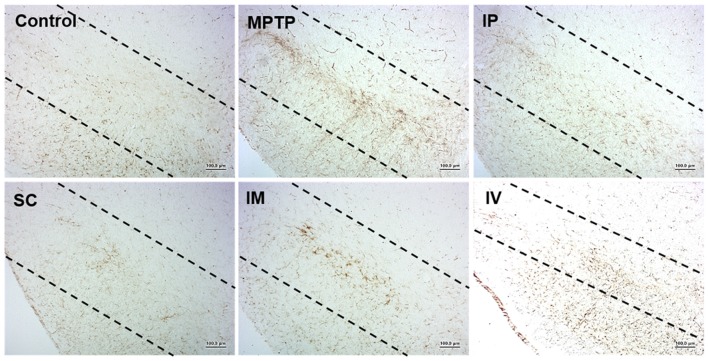
Effects of different routes of bvPLA2 administration on microglial activation in an MPTP-induced neurotoxicity model. The activation of microglia was detected by Iba-1 immunostaining. Data show that s.c. injection of bvPLA2 could inhibit the elevated levels of Iba-1. Images were obtained from the SN. Scale bars: 200 μm. IP, intraperitoneal injection; SC, subcutaneous injection; IM, intramuscular injection; IV, intravenous injection.

Microglia are the resident immunocompetent and phagocytic cells in the brain that play a neuroprotective function. Microglia are rapidly activated in response to neuronal damage and produce various potentially neurotoxic compounds under neuropathological conditions. Therefore, we analyzed the level of Iba-1 as the specific marker of microglia in the model group. Figure 5 shows that i.p., s.c., and i.v. injections of bvPLA2 dramatically reduced the number of Iba-1^+^ microglia in the brains of MPTP-induced PD mice compared to those of the MPTP group. In contrast, compared with s.c. injection, i.m. injection increased Iba-1 expression.

## Discussion

In the present study, the best route of bvPLA2 administration to prevent the temporal progression of PD pathology was investigated. The results of a previous study revealed that the i.p. injection of bvPLA2 protected dopaminergic neurons by modulating neuroinflammatory responses in an MPTP-induced PD mouse model (Chung et al., 2015). In the present study to identify the optimum route of bvPLA2 administration to ameliorate the pathophysiology of PD, as shown in the previous article, the i.p. injection of bvPLA2 showed the neuroprotective effects. However, the s.c. injection of bvPLA2 showed the best neuroprotective effects, including enhanced motor functions, decreased Th1 and Th17 polarization, increased TH-positive dopaminergic neurons and reduced number of Iba-1-positive microglia in the SN of an MPTP toxicity model.

Different routes of administration were selected to achieve either the systemic or local delivery of medications in an active form. The route of administration in pharmacology and toxicology is determined by the physical characteristics of the drug, the speed at which the drug is absorbed, and the need to bypass hepatic metabolism and achieve high concentration at particular sites (Bruno et al., 2013). For therapeutic drugs, there are various routes for drug administration, including parenteral, oral, nasal, ocular, transmucosal and transdermal injections. From a clinical point of view, it is important to consider the route of drug administration that shows the most therapeutic effects. We compared the four clinically applicable routes of injection. The results of the present study suggested that the s.c. injection of bvPLA2 was the most effective for the attenuation of MPTP-induced neurotoxicity. Subcutaneous injection is administered below the epidermis and dermis layers into the subcutaneous tissue and is easily performed with a lower risk of damage to blood vessels and other structures. Insulin and hormones are commonly administered by s.c. injections. The s.c. route is generally preferred over the i.v. route because it offers more convenience to patients, improves quality of life and reduces health care costs. These findings have significant implications toward the development of a clinical protocol for PD.

Over the past few decades, many efforts have been made to identify new drug targets focusing on CNS disease, including AD, PD and Huntington disease (Trojanowski and Hampel, 2011; Harikrishna Reddy et al., 2014; Lim et al., 2017). Despite the progress in neuroscience, numerous candidate drugs for neurodegenerative diseases have shown encouraging results *in vitro* and *in vivo*, but failed to lead to clinical trials. The glial cell line-derived neurotrophic factor (GDNF) is an effective and potent neuroprotective agent for PD. However, GDNF drug development has failed, because the neurotrophic factor does not cross the blood-brain barrier (BBB; Bartus and Johnson, 2017a,b). The small lipid-soluble drug as an index of BBB transport is only valid when the molecular weight (MW) is less than or approximately equal to 400–600 Da. The majority of the therapeutic agents are larger than the size limit and thus cannot be transported across the BBB. Because bvPLA2 is a small, 15 kDa enzyme which belongs to group III secreted PLA2, bvPLA2 cannot directly reach the brain across the BBB. The presence of activated central memory T lymphocytes has been reported at blood-cerebrospinal fluid barrier (CSF) of the choroid plexus, which act as a site of immune surveillance process that allows immune cell entry to the brain in response to acute injury of inflammation (Baruch et al., 2013). In the previous and present study, we showed the bvPLA2-mediated induction of the CD4^+^CD25^+^Foxp3^+^ Treg populations. We suggested that the neuroprotective effects of bvPLA2 treatment in MPTP-induced PD model resulted from the increased infiltration of memory T lymphocytes through choroid plexus instead of direct across the BBB.

A growing body of evidence suggests that cellular and humoral immune responses are changed in the peripheral immune system of PD patients (Dauer and Przedborski, 2003). In a previous report, the adoptive transfer of splenocytes from mice immunized with Copolymer-1 (Cop-1) suppressed microglial responses, leading to the protection of dopaminergic neurons in MPTP-intoxicated mice (Benner et al., 2004). CD4^+^ T cells isolated from Cop-1 immunized mice evoked a robust neuroprotective response (Laurie et al., 2007). In contrast, the depletion of these cells abrogated this protection. These reports represent the primary role of CD4^+^ T cells in protective responses in PD. Furthermore, Reynold et al. have reported that the adoptive transfer of CD4^+^CD25^+^ Tregs can modulate immune responses in the brain, resulting in significant neuroprotection in a PD mouse model (Reynolds et al., 2007). These authors suggested that the use of immunomodulatory strategies by inducing Treg responses attenuates neuroinflammation and inhibits dopaminergic neurodegeneration to treat PD patients.

In PD, autoreactive Th1 or Th17 cells are important for the promotion of neurodegeneration. A shift in the Th1/Th2 balance toward Th1 is closely associated with motor function. Th17 cells play critical roles in the protection against mucosal inflammation. Recent studies on the neurotoxic properties of Th17 populations have reported that the infiltration of Th17 cells into the SN can exacerbate dopaminergic neuronal loss in MPTP intoxication models (Liu et al., 2017). Consistent with this finding, increased Th1 and Th17 populations in the MPTP group indicated enhanced PD-associated inflammation. Furthermore, the decreased Th17 population in s.c. injection of bvPLA2 indicated weakened PD-associated inflammation, whereas increased Tregs in the SC group suggested the enhanced suppression of inflammation.

The present study reported an optimized route of bvPLA2 administration to prevent neuronal damage in an MPTP-induced PD mouse model. The s.c. injection of bvPLA2 represented the best route of administration among i.p., s.c., i.m., and i.v. injections. The results from the present study suggest that the s.c. route of bvPLA2 administration might have potential application for the treatment of PD patients.

## Author Contributions

HNJ and HIJ performed the experiments and conducted the statistical analysis. HBaek drafted the first version of the manuscript. HNJ and HBaek drafted sections of the manuscript. HBae supervised the project and edited the manuscript. All authors contributed to the manuscript revision and have read and approved the submitted version.

## Conflict of Interest Statement

The authors declare that the research was conducted in the absence of any commercial or financial relationships that could be construed as a potential conflict of interest.
